# Modulation of neutrophil chemotaxis by Aggregatibacter actinomycetemcomitans leukotoxin

**DOI:** 10.1080/20002297.2017.1325259

**Published:** 2017-06-09

**Authors:** Josefine Hirschfeld, Anders Johansson, Rolf Claessonand, Iain L. C. Chapple

**Affiliations:** ^a^ School of Dentistry, University of Birmingham, UK; ^b^ Department of Odontology, Section Molecular Periodontology, Umeå University,Umeå, Sweden; ^c^ Department of Odontology, Section Oral Microbiology, Umeå University, Umeå, Sweden; ^d^ Department of Periodontology, Birmingham Dental School and Hospital, Edgbaston, Birmingham, UK

## Abstract

**Objective**: Aggressive periodontitis (AgP) is frequently associated with the presence of *Aggregatibacter actinomycetemcomitans*, a Gram-negative pathogen, which expresses leukotoxin (Ltx) as a virulence factor. Ltx is known to disturb neutrophil function and to activate these cells. This study was aimed at investigating the ability of Ltx to alter the chemotactic behaviour of neutrophils, the most abundant leukocyte in periodontitis.

**Methods**: Neutrophils from healthy blood donors were isolated and stimulated or primed with low concentrations of Ltx (1, 5 and 10 ng/mL). Neutrophil migration in response to Ltx gradients and to the biofilm-derived chemotactic agent fMLP after Ltx-priming was monitored by real-time video microscopy using an Insall chamber.

**Results**: Although Ltx alone enhanced neutrophil movement at all concentrations, low directional accuracy was observed compared to the positive control (fMLP). Ltx priming led to directional movement towards fMLP with significantly enhanced speed. In case of 10 ng/mL Ltx, however, this chemotactic response towards fMLP appeared to be delayed.

**Conclusion**: The results indicate that Ltx induces non-directional neutrophil movement, whilst enhancing migration towards fMLP. Clincially, this may lead to the presence of a higher number of activated neutrophils in the periodontal tissues, potentially causing more pronounced host tissue damage.

